# Challenges in Managing a Medical School

**DOI:** 10.1590/1516-3180.2022.141216122022

**Published:** 2023-03-10

**Affiliations:** I MD, PhD. Vice Director, Faculdade de Medicina da Universidade de São Paulo HCFMUSP, São Paulo, SP, BR; Full Professor, Department of Cardiopulmonology, Instituto do Coração, Hospital das Clínicas, Faculdade de Medicina, Universidade de São Paulo, São Paulo, SP, BR. Director, Scientific Department, Associação Paulista de Medicina (APM), São Paulo, SP, BR.; Universidade de São Paulo, Faculdade de Medicina, Hospital das Clínicas, São Paulo, SP, BR; Associação Paulista de Medicina, Scientific Department, São Paulo, SP, BR; II MD, PhD. Director, Faculdade de Medicina, Universidade de São Paulo, São Paulo, SP, BR. Full Professor, Department of Clinical Medicine, Hospital das Clínicas HCFMUSP, Faculdade de Medicina, Universidade de São Paulo, São Paulo, SP, BR.; Universidade de São Paulo, Faculdade de Medicina, Hospital das Clínicas HCFMUSP, São Paulo, SP, BR

We recently had the honor and enormous challenge of taking over as co-directors of the School of Medicine of the University of São Paulo (FMUSP).

FMUSP has been nationally and internationally recognized for its pioneering spirit and excellence, both in terms of teaching and research and university extension. It has been training professionals for 110 years in the fields of medicine, physiotherapy, speech therapy, and occupational therapy, and the most recent Medical Physics Program establishes a partnership between the School of Medicine and the Physics Institute of the University of São Paulo.

Our numbers speak for themselves: we have approximately 1,400 undergraduate students; more than 1,000 employees, including 368 professors; 2,000 graduate students; and 1,600 residents. We publish more than 2,500 scientific articles annually.

Our Clinical Hospital (CH) is the largest hospital in Latin America, a state institution linked to the Secretary of Health of the State of São Paulo for administrative coordination purposes and associated with FMUSP for teaching, research, health initiatives, and community services. The CH comprises eight institutes (Central Institute, Psychiatry Institute, Heart Institute, Radiology Institute, Cancer Institute, Institute for Children and Adolescents, Orthopedics and Traumatology Institute, and Physical Medicine and Rehabilitation Institute) and two auxiliary hospitals (Suzano Auxiliary Hospital and Cotoxó Hospital Complex). Annually, we provide more than one million outpatient appointments, 180,000 urgent and emergency care appointments, and approximately 50,000 surgeries.

We are one of the largest medical-scientific research centers in Brazil, with 66 medical investigation laboratories, 230 research groups, and extensive intellectual output.

Our faculty and healthcare professionals represent a huge competitive advantage for obtaining resources in the areas of clinical research, innovation, education, and assistance. Many of our professors are among the most influential researchers in the world, according to an assessment by the British consultancy Clarivate Analytics.^1^

In the search for community-based planning that reflects the best strategies to meet the institution's needs and define its future directions, the FMUSP 2030 strategic plan was prepared in December 2021 to outline new goals, processes, and guidelines for the new cycle that has just begun. Several CH leaders and representatives from the Zerbini Foundation (ZF) and the School of Medicine Foundation (SMF) participated in it.

Planning is integrated between the FMUSP and the CH, with a focus on care, technical, and administrative teaching processes, research and innovation actions, and human resources.

The actions are developed according to strategic axes, which deal with current themes of high social relevance, such as Excellence in Teaching, Culture, and Extension; Excellence in Care; Integration; Humanization-Participatory Management; Internationalization; Sustainability; and Research, Innovation, and Entrepreneurship.

Faced with this challenge, we chose “talent retention” as our management motto. This motto represents our institution's conviction that guaranteeing the future of our school as a place of transformation and excellence depends on our greatest immaterial wealth: the employees working at the FMUSP complex.

However, “talent retention” requires economic sustainability; thus, we need to improve our capacity to retain qualified human resources, which involves competitive compensation and greater investments in infrastructure, innovation, and internationalization.

The SMF and the ZF are private, non-profit institutions that provide FMUSP with support and institutional assistance for the viable management of our hospitals, also strengthening teaching and research. We have the challenge of increasing fundraising so that we can continue investing and financing extension courses, research projects, conferences, clinical studies, technological innovation, and entrepreneurship programs, among many other initiatives.

It is with this focus on sustainability and welcoming that we intend to guarantee the excellence of our school, which today has three very solid pillars, teaching, research, and care, in addition to another pillar present in each of these axes, innovation.

We envision the need for investment in the School of Permanent Education (SPE), a unit that promotes technical, graduate, medical, and multidisciplinary training programs for the community, in addition to being a fundraising source. We intend to renew this unit so that it becomes even better recognized and more attractive to the community and to provide our employees with the possibility of improvement, with a remuneration compatible with market rates, giving them even more of a stake in our institution.

Our health complex offers one of the largest fields of professional practice in Latin America, with potential for training human resources in other areas of knowledge that remain underused. We understand that the challenges of today's world are complex and that responding to them requires dialogue between different areas of knowledge, transcending the limits of each individual profession and going beyond teamwork.

Research and innovation must be integrated as a central objective of professional training. Our students must be provided with this qualification in their training to advance science and technological development.

We need to enhance interprofessional and intersectoral training and expand the support network for undergraduate students, residents, and graduate students (pedagogical, financial, and mental health needs), while also assuring an institutional environment that respects human rights and diversity. We are also convinced that internationalization within a globalized world must be one of the missions of higher education so that we can reap the benefits of global interconnection and avoid having a limited view of our scientific role. We need more programmatic and organizational strategies to help us in this process of cooperation and international academic mobility.

We need to clearly define which health professionals we want to train, considering the enormous social investment in this school, its high qualifications, and its ability to create knowledge, not just transmit it.

Our challenges are enormous. The COVID-19 health crisis we are facing is one of them. There was an unprecedented mobilization in the face of so much adversity. FMUSP has adapted to a new reality, incorporating tools for distance learning and conferences, regrouping teams and lines of research around a single goal, adjusting and adapting all the care offered in our health units, training and qualifying collaborators, developing innovative solutions to meet care needs, in addition to many other initiatives, all of them emergencies. It was necessary to leave the comfort zone, integrate new knowledge, spread initiatives, in short, unite the “house,” to offer the population quality care and to receive more than 10,000 critically ill patients.^2^

We count on the commitment of our very talented team to working tirelessly so that “Casa de Arnaldo” continues its trajectory of excellence and pioneering spirit, training and transforming people and welcoming human beings.
